# Malignant peripheral nerve sheath tumor of the transverse colon with peritoneal metastasis: a case report

**DOI:** 10.1186/s13256-018-1896-4

**Published:** 2019-01-18

**Authors:** Gireesha Rawal, Sufian Zaheer, Charanjeet Ahluwalia, Indrani Dhawan

**Affiliations:** Department of Pathology, Vardhman Mahavir Medical College & Safdarjung Hospital, New Delhi, India

**Keywords:** Malignant peripheral nerve sheath tumor, Colon, Metastasis

## Abstract

**Background:**

Malignant peripheral nerve sheath tumors are malignant tumors arising from a peripheral nerve or displaying nerve sheath differentiation. Gastrointestinal malignant peripheral nerve sheath tumors are rare and malignant peripheral nerve sheath tumor of the colon is even rarer. To date, only five cases have been reported as malignant peripheral nerve sheath tumor arising in the colon. This is probably the first case report of malignant peripheral nerve sheath tumor of the transverse colon associated with peritoneal metastasis.

**Case presentation:**

A 25-year-old Indian man presented with a large abdominal mass. A computed tomography scan revealed a large 18 cm-sized mass in his transverse colon, suggestive of gastrointestinal stromal tumor. A wide local excision was performed. Histopathology showed sheets and fascicles of elongated to spindle-shaped tumor cells showing a moderate degree of pleomorphism and atypia. Based on morphology and immunohistochemistry, a final diagnosis of malignant peripheral nerve sheath tumor of the transverse colon was given. A peritoneal metastatic tumor deposit was identified grossly and confirmed on histopathology.

**Conclusion:**

This is a rare case report discussing the detailed diagnostic approach along with an extensive review of the literature for malignant peripheral nerve sheath tumor arising in the colon.

**Electronic supplementary material:**

The online version of this article (https://doi.org/10.1186/s13256-018-1896-4) contains supplementary material, which is available to authorized users.

## Background

Malignant peripheral nerve sheath tumors (MPNSTs) are malignant tumors arising from a peripheral nerve or displaying nerve sheath differentiation. Common sites of occurrence of MPNSTs include trunk, extremities, head, neck, and paravertebral regions. MPNSTs of the gastrointestinal tract (GIT) are rare and MPNST of the colon is even rarer.

Myenteric schwannomas are responsible for 2 to 6% of GIT stromal tumors [1], with the stomach being the most common site of occurrence [2]. Schwannomas of the colon in the absence of neurofibromatosis (NF) are exceedingly rare [2–5], with even fewer malignant cases. To the extent of our knowledge, there have been only five cases reported as MPNST arising in the colon [6–10]. This is probably the first case report of MPNST in the transverse colon associated with peritoneal metastasis, without antecedent NF or parasitic infection. Our detailed diagnostic approach and an extensive review of the literature are discussed.

## Case presentation

**Fig. 1:**
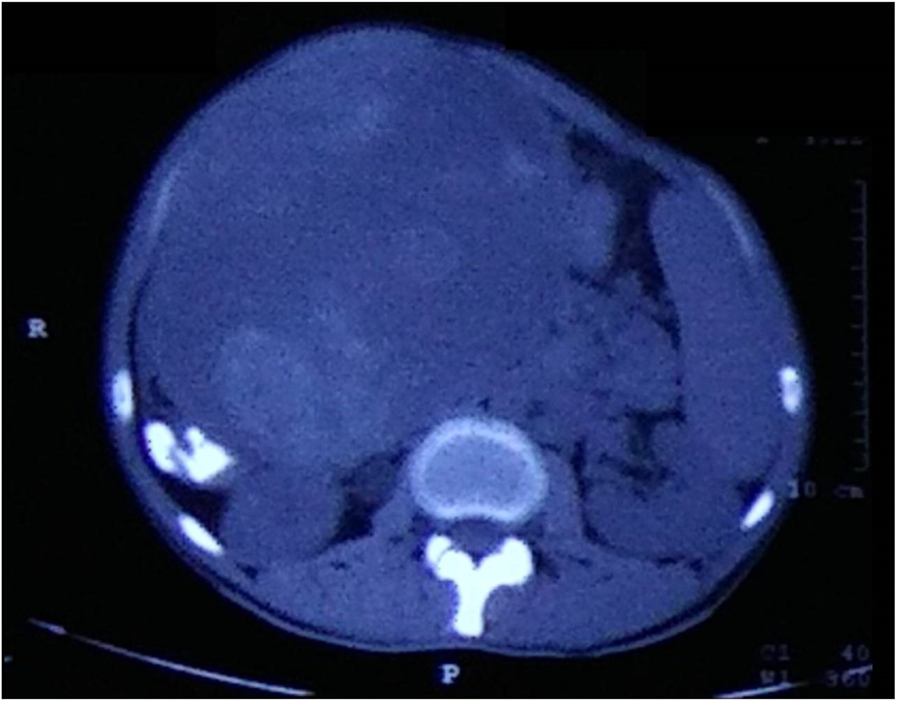
Computed tomography scan showing a large, well-circumscribed, heterogenous mass arising from the transverse colon

A 25-year-old Indian man presented with a large abdominal mass that had been increasing in size for 2 months. He complained of significant weight loss. There was no history of similar complaints or any intervention in the past. The remaining medical history, family history, and psychosocial history were unremarkable. On examination, his abdomen was hugely distended and overlying skin was unremarkable. A firm-to-hard mass could be palpated in the right lumbar, right iliac, and umbilical regions. A computed tomography (CT) scan revealed a large 18 cm-sized mass in his transverse colon, suggestive of gastrointestinal stromal tumor (GIST; Fig. 1).

**Fig. 2:**
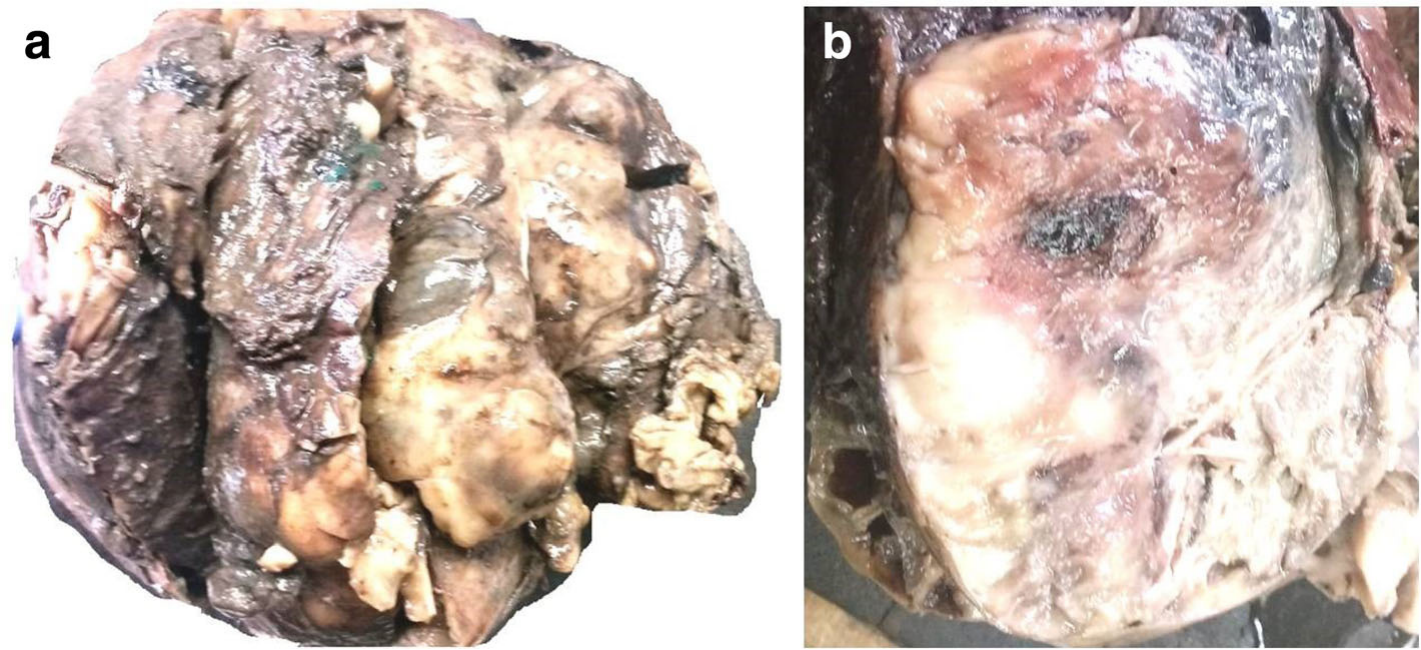
**a** Gross specimen showing a large, globular tumor. **b** Cut surface showing the presence of solid gray-white areas along with areas of hemorrhage and cystic change

A wide local excision was performed, and the specimen was sent for histopathology. On gross examination the tumor was large and globular, measuring 20 × 18 × 10 cm, and was seen to be arising from the wall of the intestine. Cut surface showed the presence of solid gray-white areas along with areas of hemorrhage and cystic change (Fig. 2).

**Fig. 3:**
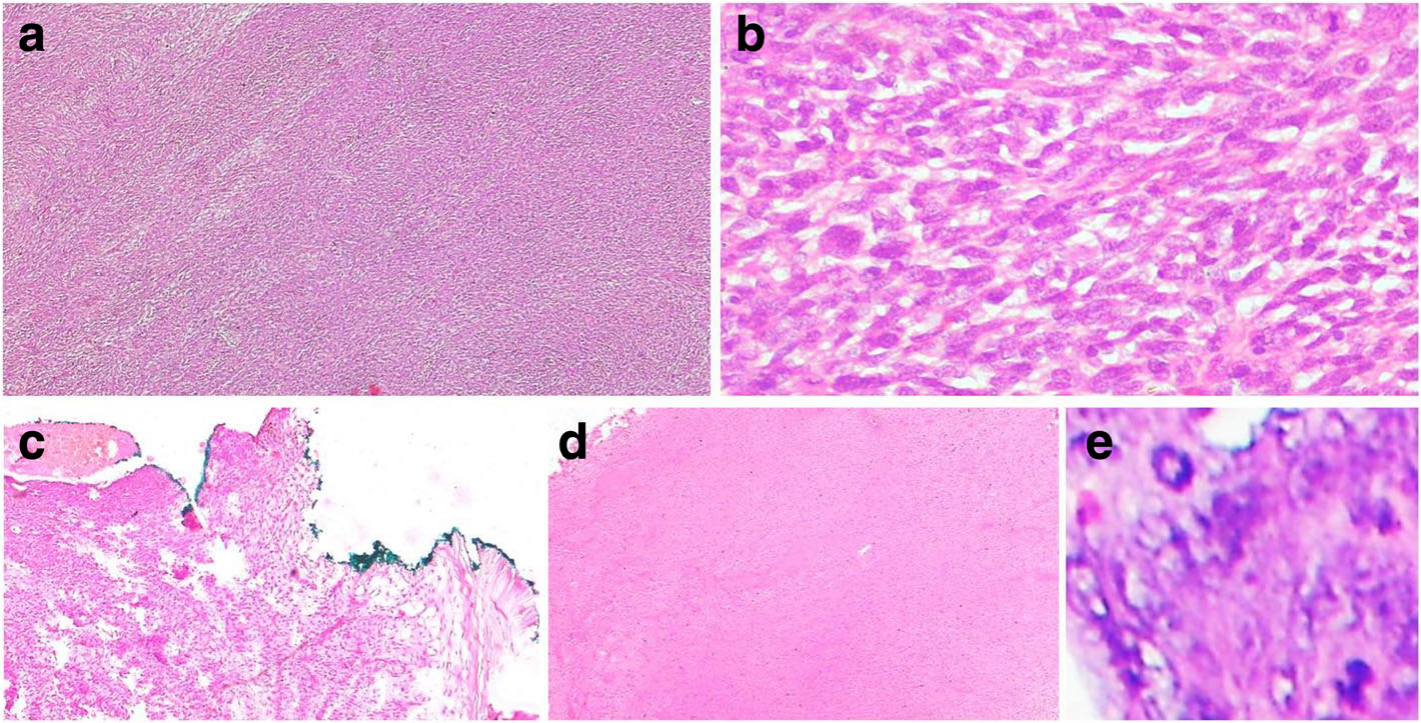
**a** Hematoxylin and eosin sections showing sheets and fascicles of elongated to spindle-shaped tumor cells (× 4). **b** Tumor cells having elongated hyperchromatic nuclei and mild to moderate amount of cytoplasm, showing moderate degree of pleomorphism and atypia (× 20). **c** Tumor reaching up to inked serosal resected margin (× 4). **d** Tumor necrosis (× 4). **e** Atypical mitosis (× 40)

Histopathology sections showed sheets and fascicles of elongated to spindle-shaped tumor cells, showing a moderate degree of pleomorphism and atypia. The individual tumor cells had elongated hyperchromatic nuclei and a mild to moderate amount of cytoplasm. Mitosis, including atypical forms, was seen. Focal areas of tumor necrosis were seen. The tumor reached up to serosal resected margin (Fig. 3).

**Fig. 4:**
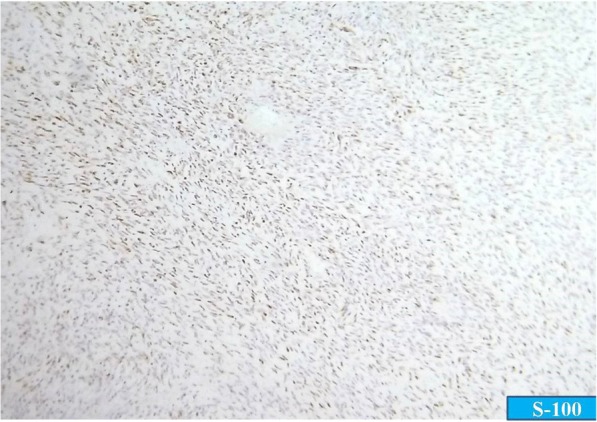
Immunohistochemistry showing patchy positivity for S-100

On morphology, a diagnosis of malignant GIST seemed to be likely, and immunohistochemistry (IHC) for CD-117, Dog-1, and CD34 was applied for confirmation. However, to our surprise, these markers came out negative. Further, the morphology was reviewed and a differential diagnosis of leiomyosarcoma and MPNST was taken into consideration, for which an IHC panel comprising smooth muscle actin (SMA) and S-100 was put up. The tumor cells showed positivity for S-100 and were negative for SMA, thus ruling out leiomyosarcoma. Again, this led to two differentials, one being gastrointestinal autonomic tumor (GANT) and the other being MPNST. However, GANT is positive for CD-117 and may be negative for S-100; even when it shows S-100 positivity, it is uniformly positive. Moreover, the patchy positivity of S-100, as was seen in our case, is characteristic of MPNST (Fig. 4).

Hence, a final diagnosis of MPNST of the transverse colon was given. The resected margins as well as resected lymph nodes were free of the tumor. However, a peritoneal metastatic tumor deposit was identified grossly, and confirmed on histopathology, signifying an advanced stage tumor, with higher stage of the neoplasm and poorer prognosis of our patient.

Postoperatively, he was discharged and advised to review with medical and radiation oncologists. He came back for the reversal of the stoma and restoration of bowel continuity. Postoperative contrast-enhanced CT (CECT) of his abdomen did not show any evidence of residual or recurrent tumor. Further, he was operated on for dismantling of the colostomy fistula, resection of 5 cm of the colon confirmed (by frozen section) to have margins negative for tumour, and colocolic anastomosis. His postoperative recovery was uneventful. The final histopathology confirmed the absence of any residual disease, thus eliminating the need for adjuvant therapy.

He has been followed up for 6 months, and is presently doing well (Additional file 1: Case timeline).

## Discussion

According to the World Health Organization (WHO), MPNSTs are defined as tumors originating from a peripheral nerve or displaying nerve sheath differentiation. They are the sixth most common type of soft tissue sarcoma [11, 12]. Sporadic origin accounts for nearly half of all MPNST cases, while other cases occur in association with NF1 [13, 14].

Common sites of occurrence of MPNSTs include trunk, extremities, head, neck, and paravertebral regions. Gastrointestinal MPNSTs are rare and MPNST of the colon is even rarer. To date only 15 cases of MPNST of GIT have been reported in the literature, out of which only five cases were seen to be arising from the colon [6–10]. Usually they occur in association with von Recklinghausen disease [9] or *Schistosoma japonicum* infection [10], neither of which was seen in the present case.

The age range of patients with MPNST of the colon varies from 2 days to 60 years in various case reports. The present case is of a 25-year-old man. None of the clinical features of MPNST of the intestine are characteristic, and most patients present with non-specific symptoms like weight loss, fatigue, pain in the abdomen, emesis, or rectal bleeding. Owing to this, preoperative diagnosis of MPNST is somewhat difficult and frequently delayed.

**Fig. 5:**
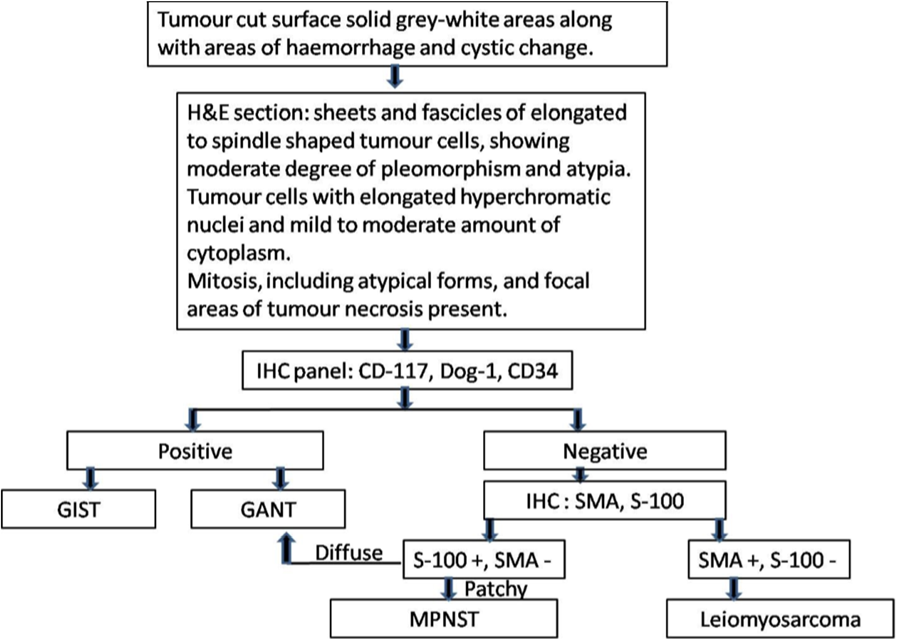
Diagnostic approach to malignant peripheral nerve sheath tumor of the colon. *GANT* gastrointestinal autonomic tumor, *GIST* gastrointestinal stromal tumor, *H&E* hematoxylin and eosin, *IHC* immunohistochemistry, *MPNST* malignant peripheral nerve sheath tumor, *SMA* smooth muscle actin

We considered malignant GIST and leiomyosarcoma as differential diagnoses. In a previous study, a majority of GISTs of the colon were positive for c-kit and CD34 but none of them showed desmin or S-100 positivity. On the contrary, leiomyosarcomas of the colon were all negative for CD34 and c-kit but most of them showed positivity for SMA, desmin, or both [15]. In our case, we observed patchy positivity for S-100 protein and negativity for CD-117, Dog1, CD34, and SMA. These IHC findings are in contrast to the findings expected in GIST or smooth muscle tumors (Fig. 5).

In cases of soft tissue MPNST, 40 to 65% of patients encounter local recurrence, while metastases occur in 30 to 60% of cases within 1 year of surgery [16]. Factors predicting recurrence comprise tumor site, size (≥ 10 cm), and adequacy of surgical margins. Factors that predict metastases include tumor size (≥ 10 cm) and stage (American Joint Committee on Cancer stage III) [16]. The site involved by metastases in most of the cases (> 2/3) is lungs, although other sites including liver, brain, bone, or adrenal gland may also be involved [16]. Presently, little is known about intestinal MPNST, which is thought to have an even more adverse prognosis than its soft tissue analog. Only one case report describes metastasis in MPNST of the small intestine, with the site being peritoneal metastasis, as in our case [17].

The optimal treatment of MPNST of the colon is not well established, owing to its rare occurrence [7]. The current recommendations and treatment are based on knowledge of this tumor at other sites. The current standard of care for localized MPNST is complete surgical resection with wide negative margins, which is a strong predictor of survival [16]. The role of adjuvant therapy is controversial. Adjuvant radiotherapy is recommended when clear surgical resection margins are not possible, that is, for local control [18]. However, MPNST of the colon may not benefit from radiotherapy due to its location in the abdominal cavity, where radiotherapy may lead to stricture formation and restricted intestinal mobility [9]. Only a few studies have probed the role of chemotherapy for treatment of MPNSTs. However, a recent meta-analysis of the pooled data of 12 trials examining the efficacy of first-line chemotherapy in the treatment of MPNSTs reported promising results with the combination of doxorubicin and ifosfamide [19]. Recently target therapy using molecular pathways in MPNST has been developed, but further studies are warranted [16, 20]. Hence, proper management of such tumors requires a multidisciplinary approach.

## Conclusion

This is probably the first case report of MPNST of the transverse colon with peritoneal metastasis that discusses the detailed diagnostic approach and has an extensive review of the literature. Owing to its non-specific presentation, a preoperative diagnosis of MPNST is somewhat difficult and frequently delayed. However, in a patient with a mesenchymal tumor, the possibility of MPNST should always be considered, even at unusual sites like the colon. This diagnosis has substantial significance since the prognosis of patients who develop MPNST of the colon has been considered grave compared to that of patients with other mesenchymal tumors of the colon (for example, GIST or leiomyosarcoma) as well as that of patients with soft tissue MPNST. The optimal treatment of MPNST of the colon is not well established owing to its rare occurrence. The current standard of care for MPNST is complete surgical resection with wide negative margins, with the role of chemotherapy and radiotherapy still under evaluation. Proper management of such tumors requires a multidisciplinary approach.

## Additional file

Additional file 1: Case timeline. (PDF 276 kb)

## Abbreviations

CECT: Contrast-enhanced computed tomography
CT: Computed tomography
GANT: Gastrointestinal autonomic tumor
GIST: Gastrointestinal stromal tumor
GIT: Gastrointestinal tract
IHC: Immunohistochemistry
MPNST: Malignant peripheral nerve sheath tumor
NF: Neurofibromatosis
SMA: Smooth muscle actin
WHO: World Health Organization
