# Supplementary material for: Malignant peripheral nerve sheath tumor of the transverse colon with peritoneal metastasis: a case report
Source: J Med Case Rep. 2019 Jan 18;13:15. doi: 10.1186/s13256-018-1896-4 (PMC6337829; doi:10.1186/s13256-018-1896-4)
Supplement: Supplementary file 1 — Case timeline. (PDF 276 kb) [file 13256_2018_1896_MOESM1_ESM.pdf]

No history of similar complaints or any intervention in the past. The remaining medical history, family history and psychosocial history were unremarkable

Current Illness: 25 years old male patient presented with large abdominal mass, increasing in size since 2 months. Significant weight loss.

Physical examination: abdomen hugely distended, overlying skin was unremarkable. Firm to hard mass palpated in right lumbar, right iliac and umbilical regions

Diagnostic Evaluations: CT scan revealed large mass in transverse colon.

On biopsy differential diagnosis were GIST, GANT, MPNST.

IHC confirmed MPNST.

Final Diagnosis: MPNST, transverse colon with peritoneal metastasis.

Initial Treatment: Wide local excision

Referral: medical and radiation oncologists

Postoperative CECT abdomen no evidence of residual or recurrent tumour.

Dismantling of colostomy fistula, resection of 5 cm of colon confirmed (by frozen section) to have margins negative for tumour & colocolic anastomosis.

Final histopathology confirmed absence of any residual disease, thus eliminating need for adjuvant therapy.

Final follow up: Presently doing well.

Resolution of this episode of care
